# CRISPR-Cas9 assisted non-homologous end joining genome editing system of *Halomonas bluephagenesis* for large DNA fragment deletion

**DOI:** 10.1186/s12934-023-02214-y

**Published:** 2023-10-14

**Authors:** Chunyan Liu, Yaxin Yue, Yanfen Xue, Cheng Zhou, Yanhe Ma

**Affiliations:** 1 State Key Laboratory of Microbial Resources, Institute of Microbiology, Chinese Academy of Sciences, Beijing, 100101 China; 2 University of Chinese Academy of Sciences, Beijing, 100049 China; 3 College of Biochemical Engineering, Beijing Union University, Beijing, 100023 China; 4 Beijing Key Laboratory for Utilization of Biomass Wastes, Beijing, 100023 China

**Keywords:** *Halomonas*, CRISPR, NHEJ, Gene knockout, Large fragment deletion

## Abstract

**Background:**

Halophiles possess several unique properties and have broad biotechnological applications including industrial biotechnology production. *Halomonas* spp., especially *Halomonas bluephagenesis*, have been engineered to produce various biopolyesters such as polyhydroxyalkanoates (PHA), some proteins, small molecular compounds, organic acids, and has the potential to become a chassis cell for the next-generation of industrial biotechnology (NGIB) owing to its simple culture, fast growth, contamination-resistant, low production cost, and high production value. An efficient genome editing system is the key for its engineering and application. However, the efficiency of the established CRISPR-Cas-homologous recombination (HR) gene editing tool for large DNA fragments was still relatively low. In this study, we firstly report a CRISPR-Cas9 gene editing system combined with a non-homologous end joining (NHEJ) repair system for efficient large DNA fragment deletion in *Halomonas bluephagenesis*.

**Results:**

Three different NHEJ repair systems were selected and functionally identified in *Halomonas bluephagenesis* TD01. The NHEJ system from *M. tuberculosis* H37Rv (Mt-NHEJ) can functionally work in *H. bluephagenesis* TD01, resulting in base deletion of different lengths for different genes and some random base insertions. Factors affecting knockout efficiencies, such as the number and position of sgRNAs on the DNA double-strands, the Cas9 protein promoter, and the interaction between the HR and the NHEJ repair system, were further investigated. Finally, the optimized CRISPR-Cas9-NHEJ editing system was able to delete DNA fragments up to 50 kb rapidly with high efficiency of 31.3%, when three sgRNAs on the Crick/Watson/Watson DNA double-strands and the arabinose-induced promoter P_ara_ for Cas9 were used, along with the background expression of the HR repair system.

**Conclusions:**

This was the first report of CRISPR-Cas9 gene editing system combined with a non-homologous end joining (NHEJ) repair system for efficient large DNA fragment deletion in *Halomonas* spp. These results not only suggest that this editing system is a powerful genome engineering tool for constructing chassis cells in *Halomonas*, but also extend the application of the NHEJ repair system.

**Supplementary Information:**

The online version contains supplementary material available at 10.1186/s12934-023-02214-y.

## Background

*Halomonas bluephagenesis* TD01 is a moderately halophilic bacterium that can tolerate salt concentration of 10–250 g/L. It can achieve continuous fermentation without sterilization in a high salt concentration growth environment, using seawater for fermentation, which reduces the consumption of fresh water resources [1]. The fermentation tank can be made of plastic, ceramic or even cement, which reduces the costs of equipment. Moreover, its metabolites are rich in polyhydroxyalkanoates (PHA), a degradable material with multiple applications. Therefore, *H. bluephagenesis* TD01 has the advantage of becoming “the chassis cell of next generation of industrial production” [2]. Efficient genetic manipulation technology including gene editing technology is essential for microbial chassis cell construction and industrial applications. CRISPR gene editing technology as an efficient, accurate and cost-effective gene editing technology has been successfully and widely used for gene editing of microbial cells. The development of CRISPR gene editing technology in *H. bluephagenesis* TD01 is conducive to accelerating its optimization of the metabolic pathway and construction of chassis cells. Qin et al. established CRISPR-Cas9-homologous recombination (HR) repair gene editing technology that can delete genes in *H. bluephagenesis* TD01 [3]. However, this gene editing system cannot delete large gene fragments (> 10 kb). And when the gene edited single colony was verified by colony PCR, it was found that there would be a mixed type, i.e., the single colony had both a successful and unsuccessful knock-out. Recently, although Xu et al. optimized this gene editing tool for deleting large DNA fragments, its efficiency was still relatively low (approximately 12.5% for 50 kb) [4]. This meant that these gene editing technologies were not efficient and could not meet the need for large fragment deletions for the construction of chassis cells of *H. bluephagenesis*. Therefore, it is necessary to develop an efficient, convenient and untraceable gene editing system.

To date, the repair systems of the combined CRISPR gene editing system are mainly the HR repair system and the non-homologous end joining (NHEJ) repair system [5, 6]. The process of repairing double-strand breaks (DSBs) using NHEJ repair system in prokaryotes is similar to that in eukaryotes. However, only few protein types are involved in prokaryotic NHEJ repair system, mainly Ku and LigD proteins. During DNA repair, the Ku protein, which is present in many bacteria such as *Actinomycetes*, *Bacillus*, *Agrobacterium* and *Methanosarcina* [7–13], generally binds to DSBs but does not interact with closed circular DNA because it first recognizes DNA ends and then transfers them to internal sites [14]. The LigD protein, an ATP-dependent ligase also found in strains containing the Ku protein, such as YkoU, BH2209 (*Bacillus halodurans*), Rv0938 (*Mycobacterium tuberculosis*), PA2150 and AF-1725 (*Archaeoglobus fulgidus*) [15], has multifunctional domains in the repair of DSBs, such as ligase, polymerase, and phosphatase domains. The repair process is mainly divided into three steps [16]: binding of Ku protein to DSBs, end processing, and ligation of DSBs. After DSBs formed, the Ku protein binds to the end of the DSBs and wraps the DSBs in a loop to avoid further degradation. At the same time, the Ku protein recruits the LigD protein, which repairs DSBs due to its multifunctional domain and a variety of ATP-dependent DNA ligase, polymerase, and nuclease activity, that can simultaneously complete the cleavage and joining of DSBs. Compared with the HR repair system, the NHEJ repair system in prokaryotes has simple components and can complete the repair of DSBs in a short time without the involvement of sister chromatids; however, this leads to inaccurate repair.

CRISPR-Cas/Cpf1 in combination with the NHEJ repair system has been used in *Escherichia coli*, *Actinomycetes*, and *Methanosarcina* for genome editing. The CRISPR-Cas9 (Cas9 protein from *Streptococcus pyogenes*) gene editing system assisted NHEJ repair system from *M. tuberculosis*, *Mycobacterium smegmatis* and *Bacillus subtilis* were successfully used for gene deletion in *E. coli* [17–19]. The longest deleted fragment reached to 123 kb, with an efficiency of 36% [19]. For *Actinomycetes*, CRISPR-Cas9/Cpf1 (Cas9 from *S. pyogenes* and Cpf1 from *Francisella novicida*) combined with endogenous NHEJ repair system from *M. tuberculosis* and *M. smegmatis* were mainly used in *Streptomyces* and *Mycobacterium* for DNA fragment deletion [20–23]. And up to 28 kb could be deleted with efficiency of up to 10%. Nayak and Metcalf found that CRISPR-Cas9 of *S. pyogenes* can be used to knock out target genes in *Methanosarcina acetivorans* using the NHEJ repair system from *Methanocella paludicola* [12]. The deleted fragments were 75–2700 bp when the NHEJ repair system repaired DSBs, which usually occurred in the microhomology region (between 6 and 11 bp), suggesting that the NHEJ repair system mediates the occurrence of microhomology mediated end joining.

Due to the great advantages for large DNA fragment deletion, in this study we successfully established a CRISPR-Cas9-NHEJ gene editing technology in *H. bluephagenesis* TD01. By further optimization, the final system can delete gene fragments up to 50 kb, laying the foundation for the construction of the smallest genome and speeding up the process of chassis cell construction.

## Results

### Selection of a suitable NHEJ repair system for ***H. bluephagenesis*** TD01

**Fig. 1:**
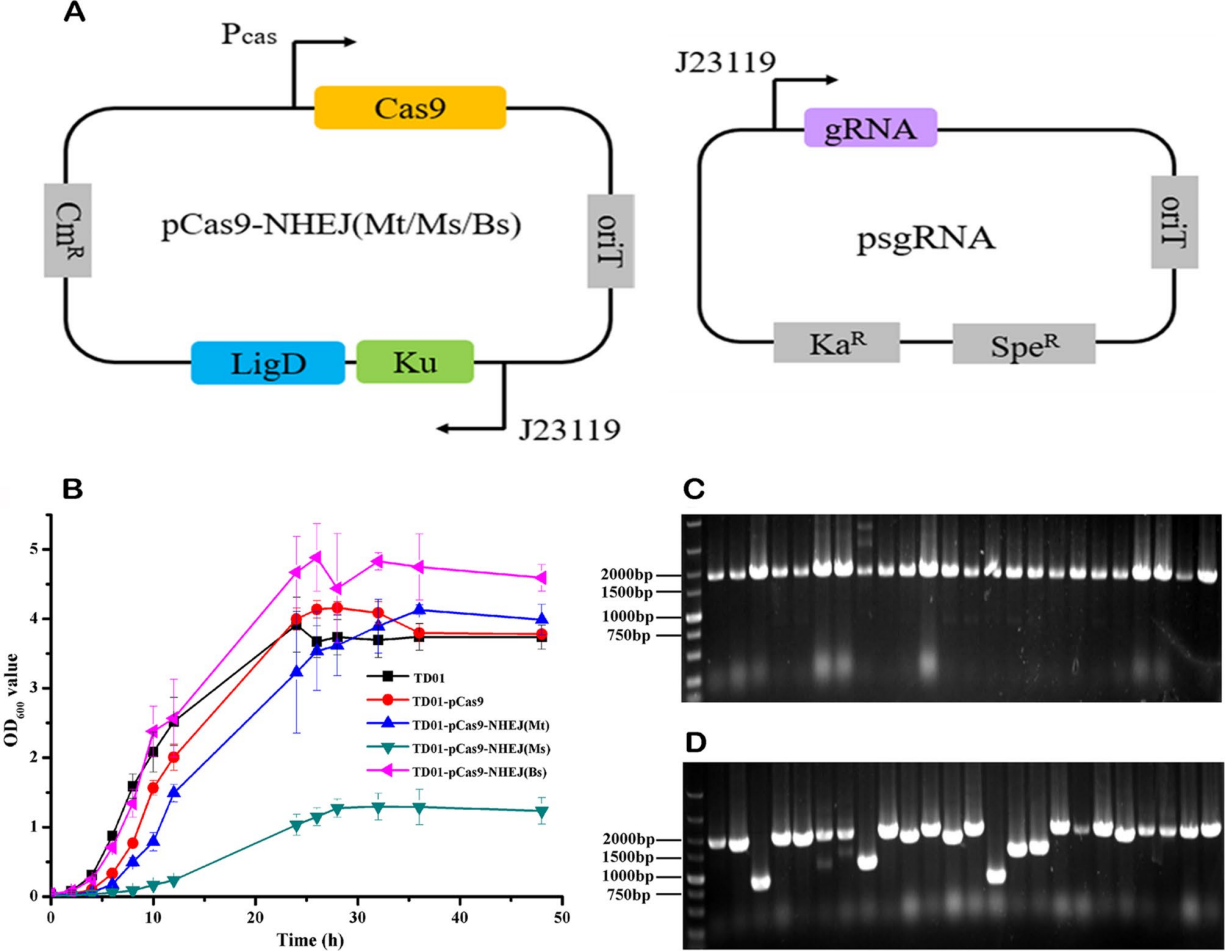
CRISPR-Cas-NHEJ gene editing system construction and repairing adaptability. (**A**) Two plasmids system for CRISPR-Cas-NHEJ gene editing system construction. The pCas9-NHEJ (Mt/Ms/Bs) and psgRNA are low copy and high copy plasmid, respectively. The promoter J23119 and P_cas_ are constitutive promoters. (**B**) Effect of Mt/Ms/Bs-NHEJ system on the growth of *H. bluephagenesis* TD01. (**C**) and (**D**) The Colony PCR results (agarose gel electrophoresis) of randomly selected clones for *phaC* deletion under the action of Bs-NHEJ and Mt-NHEJ repair system, respectively. The PCR band of wild type *phaC* gene was around 1851 bp. The PCR band less than the wild type indicate deletion of the gene segment

In order to obtain an NHEJ repair system suitable for *H. bluephagenesis* TD01, we selected three different NHEJ repair systems from *M. tuberculosis* H37Rv (Mt-NHEJ), *M. smegmatis* mc2155 (Ms-NHEJ) and *B. subtilis* 168 (Bs-NHEJ) (Table 1), which were well studied and successfully used in *E. coli* for gene deletion [17–19]. The CRISPR-Cas9-NHEJ gene editing system was constructed using two plasmids, pCas9-NHEJ and psgRNA (Fig. 1A). To confirm the feasibility of the systems, the effect of the NHEJ repair systems on the growth of *H. bluephagenesis* TD01 was tested first. The results showed that the Mt-NHEJ and Bs-NHEJ repair systems did not affect the growth of *H. bluephagenesis* TD01, whereas the Ms-NHEJ repair system inhibited growth, indicating that this system cannot be used in *H. bluephagenesis* TD01 (Fig. 1B). After that, the repairability of Mt-NHEJ and Bs-NHEJ repair systems was verified by deleting *phaC*, a gene associated with PHA synthesis in *H. bluephagenesis* TD01. A sgRNA targeted to the middle of *phaC* gene was designed and used (Table S1). Twenty-three clones of each experiment were randomly selected for colony PCR (primes were shown in Table S2). The results showed that the PCR bands of all twenty-three clones was the same as that of wild type for CRISPR-Cas9-NHEJ (Bs) system (Fig. 1C), whereas eight clones which PCR bands were smaller than that of wild type for the CRISPR-Cas9-NHEJ (Mt) system (Fig. 1D). By sequencing, the eight clones showed deletion length of 250–800 bp. These results indicated that no gene deletion was found for CRISPR-Cas9-NHEJ (Bs) system, but the CRISPR-Cas9-NHEJ (Mt) system could delete the *phaC* gene. To further confirm the repairability of Bs-NHEJ system, more 100 clones were selected for colony PCR, and still no gene deletion was found. This indicates that the Mt-NHEJ repair system can functionally work in *H. bluephagenesis* TD01, resulting in the removal of variable length bases, whereas the Bs-NHEJ repair system cannot work effectively in *H. bluephagenesis* TD01, might because of its incompatibility or low efficiency in the cells. Therefore, the Mt-NHEJ repair system with a CRISPR-Cas9 gene editing system was selected for the deletion of *H. bluephagenesis* TD01 genes.

**Table 1:** NHEJ repair system and its main components

Protein gene	*Mycobacterium* *tuberculosis* H37Rv	*Mycobacterium smegmatis* mc2 155	*Bacillus subtilis* 168
*Ku*	Rv0937c	MSMEG_5580	ykoV
*ligD*	RV0938	MSMEG_5570	ykoU

### Repair characteristics of the Mt-NHEJ repair system in ***H. bluephagenesis*** TD01

To characterize the repair properties of the Mt-NHEJ repair system in *H. bluephagenese* TD01, five additional genes of different lengths and positions were also selected for deletion using sgRNA target in the middle of gene fragments (Table S1). For each gene deletion experiment, one hundred individual colonies were randomly selected for testing using colony PCR (the primer pairs were shown in Table S2) and the positive clones (the PCR band smaller than wild type) were sequenced. And three parallel deletion experiments for each gene (totally 300 clones were selected and identified) were conducted. The results were shown in Table 2. When the Mt-NHEJ repairs DSBs, different genes may have base deletion of different lengths. The delete length of *phaC*, *gabD3*, *rfbD1*, *rfbD2*, and TD01-01921-23 was 45–1107 bp, 86–901 bp, 13–519 bp, 22–632 bp, and 10–2281 bp, respectively. In addition, the selected sgRNA showing two matches in the gene TD01-00059-61 fragment resulted in larger fragments deletion (1077–3887 bp). Except for TD01-00059-61, the proportion of colonies with deletion length longer than 1000 bp was around 3%, suggesting that the deletion length of the Mt-NHEJ repair system by single sgRNA may generally be 10–1000 bp when repairing DSB. In a few cases, the repair system also introduced insertions with different base lengths, with the maximum inserted length being 563 bp for *rfbD2*. Different average deletion efficiencies were also observed for different genes, ranging from 6.3 to 33.3%. Compared with the HR repair system, the Mt-NHEJ repair system was unfaithfully, might mainly because homology arms were not introduced (independent of the repair template) and the LigD protein had multifunctional activity during the repair process.

**Table 2:** Results of gene deletion guided by single sgRNA

Target gene (length)	Average deletion efficiency	Delete length (bp)	Insert length (bp)
*phaC* (1851 bp)	12.3 ± 2.5%	45–1107	51–87
*gabD3* (1446 bp)	6.3 ± 1.5%	86–901	30–55
*rfbD1* (897 bp)	10.0 ± 2.0%	13–519	29–144
*rfbD2* (891 bp)	33.3 ± 5.5%	22–632	2–563
TD01-01921-23 (2484 bp)	7.7 ± 0.6%	10–2281	/
TD01-00059-61 (2961 bp)	24.0 ± 1.0%	1077–3887^*a*^	14–176

^*a*^ TD01-00059-61 is a repeat sequence and the selected sgRNA has two matches in this gene

### Deletion of large gene segments by the CRISPR-Cas9-NHEJ(mt) system

**Fig. 2:**
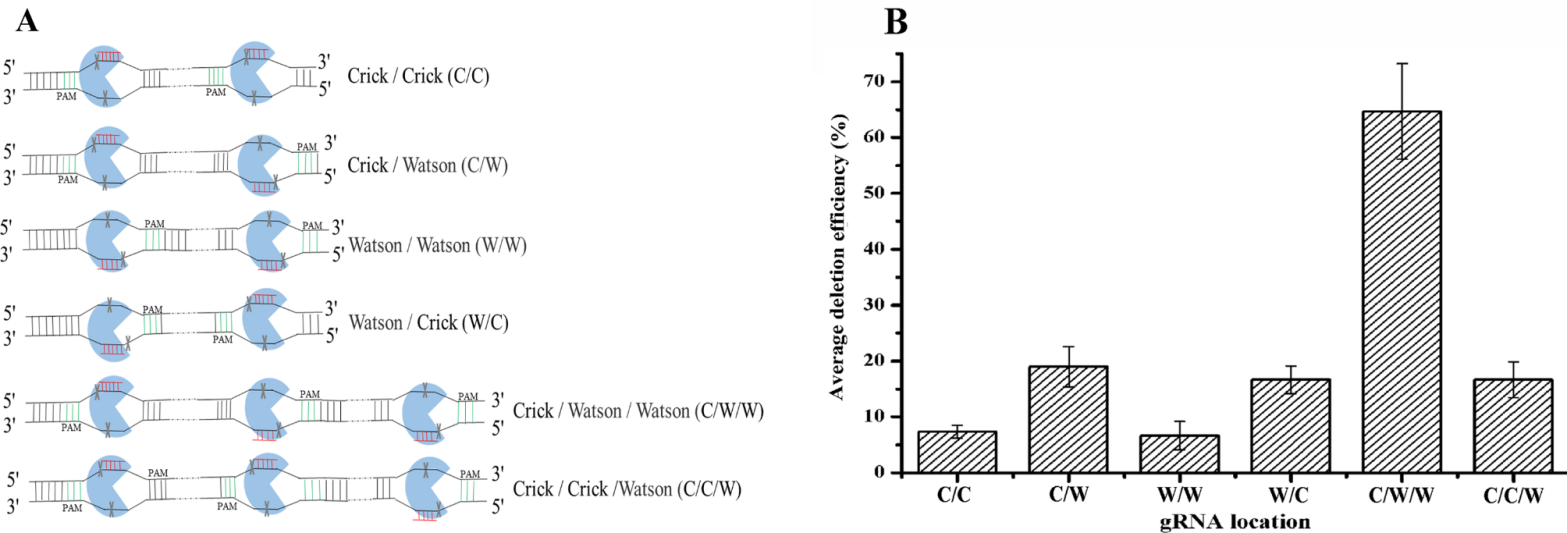
Optimization of sgRNAs number and location. (**A**) Diagrammatic sketch of different position combinations of two and three gRNAs. Blue represents Cas9 protein; green represents PAM sequence; red represents gRNA; Watson is template strand; Crick is coding strand. (**B**) Deletion efficiency of *phaC* gene guiding by two and three sgRNAs with different position combinations. For each experiment of *phaC* deletion efficiency determination, fifty clones were randomly selected for colony PCR and all experiments were performed in triplicates. The deletion efficiency was calculated by dividing the number of positive deletion clones by the total number of selected clones

The Mt-NHEJ repair system could repair DSBs in *H. bluephagenesis* TD01, therefore, we tried to delete larger fragments. The DNA fragments located at the 34,826–56,496 bp position of the *H. bluephagenesis* TD01 genome which contained multiple hypothetical genes were selected for targeting deletion. If the deletion was successful, it not only proved that the CRISPR-Cas9-NHEJ (Mt) gene editing system could delete large gene segments, but also proved that the hypothetical genes of the gene segment were non-essential genes, laying a foundation for the construction of a minimal genome. Normally, at least two sgRNAs are required for the deletion of large fragments; therefore, the location of sgRNAs on the DNA double strands was considered to be important for deletion efficiency. There are four position combinations of duplicated sgRNAs on the DNA strand for two sgRNAs, the Watson/Watson strand (W/W), Watson/Crick strand (W/C), Crick/Watson strand (C/W), and Crick/Crick strand (C/C) (Fig. 2A) [23]. The best position combination of two sgRNAs for deletion of DNA fragment was evaluated by deleting *phaC*. The two sgRNAs targeted near to both ends of gene segment were used (Table S1). Fifty clones were randomly selected for colony PCR and all experiments were performed in triplicates. The PCR products smaller than the wild type indicate the positive deletion. The results showed that when two sgRNAs with the combination of C/W and W/C was used, the deletion efficiency of *phaC* was approximately three times that of C/C and W/W (Fig. 2B). In addition, the deletion efficiency of C/W was approximately 1.2 times that of W/C. This shows that the C/W combination had the highest deletion efficiency among the four position combinations.

This combination was then used to design two sgRNAs targeted near to both ends of DNA fragments (Table S1) for deletion of 4 kb (52,204–56,496 bp), 7 kb (49,199–56,507 bp), and 9 kb (49,407–58,544 bp) fragments. For each experiment, one hundred individual colonies were randomly selected for testing by colony PCR (primer pairs were shown in Table S2) and the positive deletion clones were sequenced. The results showed that the deletion efficiency of 4 kb, 7 kb, and 9 kb was 81%, 74%, and 87%, respectively; however, random deletion of fragments also occurred (Table 3). The length of the deleted fragment was 3–21 kb, 5–20 kb and 7–21 kb when the 4 kb, 7 kb and 9 kb fragments were targeted for deletion, respectively. However, when 15 kb (41,405–56,496 bp) and 18 kb (38,533–56,496 bp) fragments were deleted by two sgRNAs (Table S1), the deletion efficiency was only 4% and 2%, with deletion length of approximate 15–17 kb and 18–19 kb, respectively (Table 3). This indicated that this genome editing system still displayed low efficiency for deletion of DNA fragments more than 9 kb.

**Table 3:** The deletion results of 4–18 kb fragments

Deletion fragment	Delete length (bp)	Colony number	Deletion efficiency
4 kb (52,204–56,496 bp)	Wild type	19	81%
	3000–5000	24	
	5000–7000	18	
	7000–10,000	9	
	10,000–15,000	20	
	15,000–20,000	7	
	>20,000	3	
7 kb (49,199–56,507 bp)	Wild type	26	74%
	<6000	1	
	6000–8000	30	
	8000–10,000	23	
	10,000–15,000	8	
	15,000–20,000	12	
9 kb (49,407–58,544 bp)	Wild type	13	87%
	7000–9000	8	
	9000–10,000	35	
	10,000–15,000	23	
	15,000–20,000	19	
	>20,000	2	
15 kb (41,405–56,496 bp)	Wild type	96	4%
	15,000–17,000	4	
18 kb (38,533–56,496 bp)	Wild type	98	2%
	18,000–19,000	2	
18 kb (38,533–56,496 bp)^*a*^	Wild type	86	14%
	18,000–20,000	14	

^*a*^ Three sgRNAs with C/W/W combination were used

### Optimization of the sgRNA number and position

The CRISPR-Cas9-NHEJ (Mt) gene editing system mainly contains three elements: sgRNA, Cas9 protein, and the Mt-NHEJ repair system. To improve the deletion ability of the CRISPR-Cas9-NHEJ (Mt) editing system for larger DNA fragments, three sgRNAs were designed. The *phaC* gene deletion efficiency was also first used to confirm the best combination. Besides the two sgRNAs with C/W combination used before, the third sgRNA was designed to target to the middle of *phaC* gene (Table S1), so that the combination of three sgRNAs positions was C/C/W or C/W/W (Fig. 2A). The results showed that the C/W/W combination had the highest deletion efficiency of the *phaC* gene, which was approximately 3.3 times that of two sgRNAs with C/W combination and 3.6 times that of three sgRNAs with C/C/W combination, respectively (Fig. 2B). Then, the experiments for deletion of the 18 kb fragments guiding by three sgRNAs (Table S1) with C/W/W combination was also conducted. One hundred individual colonies were randomly selected for testing by colony PCR and sequencing. The results showed that an increased number of sgRNAs can improve the deletion efficiency of 18 kb fragment from 2 to 14% with deletion length of approximate 18–20 kb (Table 3). Guided by three sgRNAs (Table S1), a 19 kb fragment (36,955–56,496 bp) and 26 kb fragment (692,569–718,967 bp) deletion were also performed. However, only one clone among one hundred selected clones showed deletion of about 22 kb, indicating an efficiency of only 1% for the 19 kb fragment, whereas no deletion was detected for the 26 kb fragment. This indicated that the constructed gene editing system was unable to delete large DNA fragments larger than 19 kb yet even three designed sgRNAs were used. Therefore, the CRISPR-Cas9-NHEJ (Mt) gene editing system needs further improvement.

### The relationship between Mt-NHEJ and HR repair system in ***H. bluephagenesis*** TD01

**Fig. 3:**
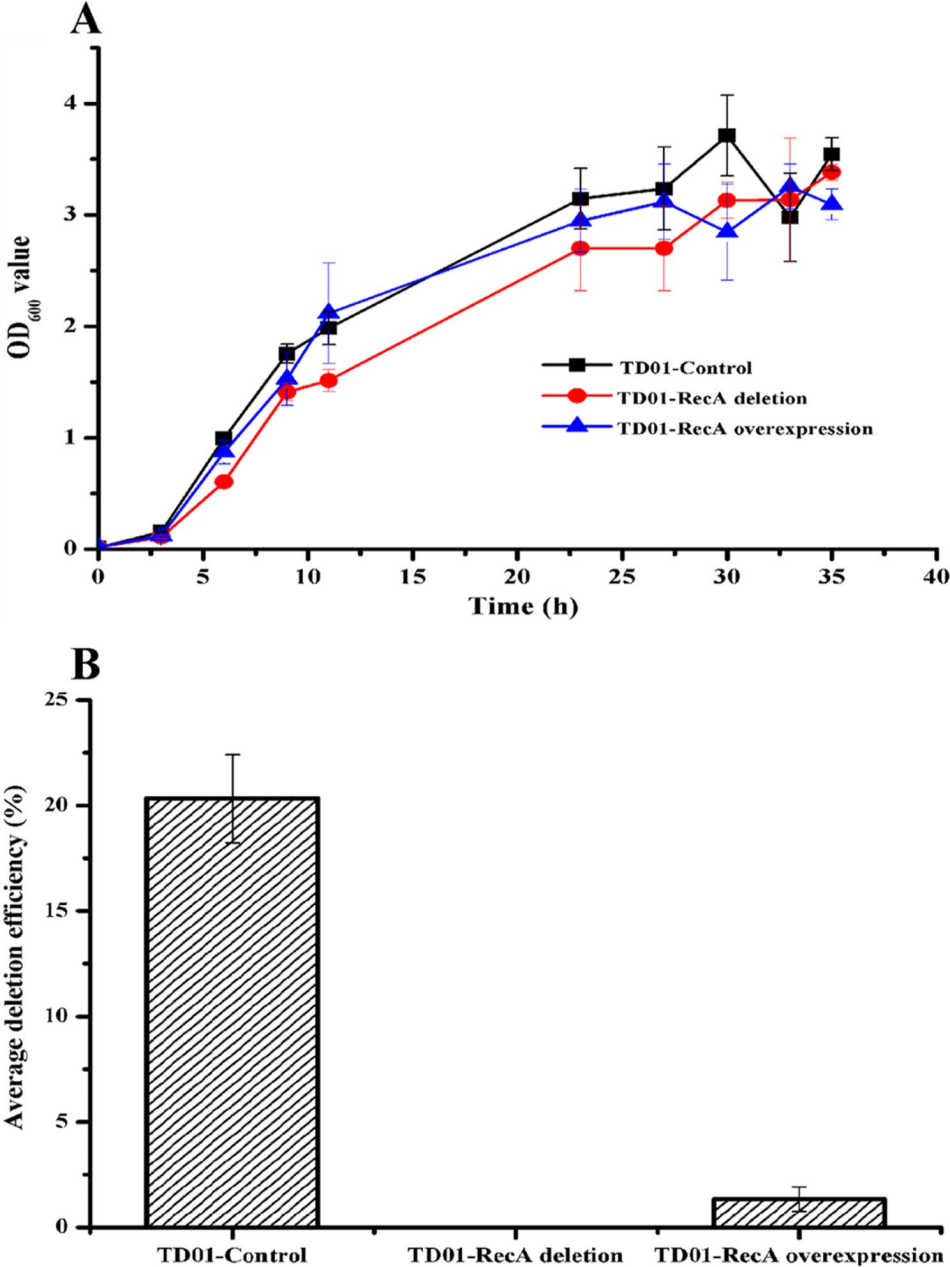
The influence of HR system on gene editing effiency of Mt-NHEJ system in *H. bluephagenesis* TD01. (**A**) Growth of *H. bluephagenesis* TD01 when *recA* was deleted and overexpressed. (**B**) Deletion efficiency of *phaC* gene guided by two sgRNAs when deletion and overexpression of *recA*. The background expression of *recA* was used as control. For each experiment of *phaC* deletion efficiency determination, fifty clones were randomly selected for colony PCR and all experiments were performed in triplicates. The deletion efficiency was calculated by dividing the number of positive deletion clones by the total number of selected clones

When the CRISPR-Cas9-NHEJ (Mt) gene editing system was introduced, there were actually two repair systems (Mt-NHEJ and HR) in *H. bluephagenesis* TD01. Previous studies have shown that the HR system can affect the repair efficiency of the NHEJ system [23]. To investigate the relationship between HR and Mt-NHEJ in *H. bluephagenesis* TD01, the endogenous HR repair system was strengthened or weakened by regulating the expression of the RecA protein, one of the key proteins of the HR repair system [24]. The *phaC* gene deletion efficiency guided by two sgRNAs was used to evaluate the effects of HR system on NHEJ system. The results showed that overexpression by the strong endogenous promoter J23119 or knockout of *recA* did not affect the growth of *H. bluephagenesis* TD01 (Fig. 3A). However, the repair activity of the Mt-NHEJ repair system was almost completely inhibited under these two conditions (Fig. 3B). This suggests that the Mt-NHEJ repair function requires the HR system, but there may be a certain relationship between the Mt-NHEJ and HR systems in *H. bluephagenesis* TD01, that differs from previous report [23] and needs further investigation.

### Optimization of Cas9 expression in the CRISPR-Cas9-NHEJ system

**Fig. 4:**
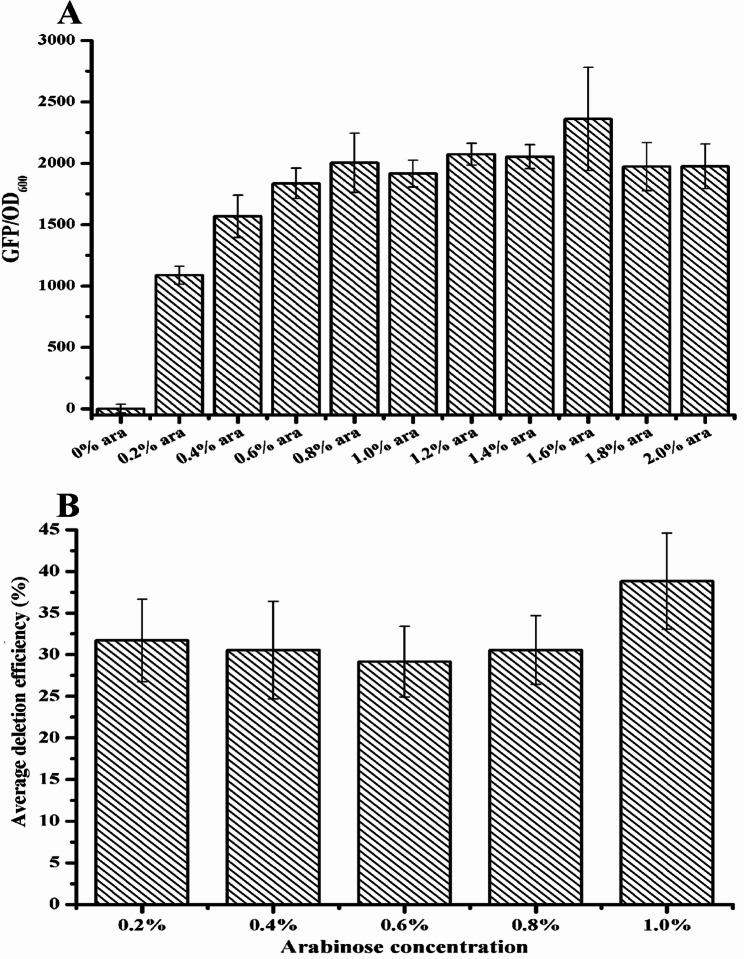
Optimization of Cas9 expression in *H. bluephagenesis* TD01. (**A**) The GFP fluorescence intensity induced by different concentrations of arabinose. (**B**) Deletion efficiency of *phaC* gene by two sgRNAs when Cas9 protein was induced by P_ara_ under different arabinose concentration. For each experiment of *phaC* deletion efficiency determination, fifty clones were randomly selected for colony PCR and all experiments were performed in triplicates. The deletion efficiency was calculated by dividing the number of positive deletion clones by the total number of selected clones

In addition to the sgRNA and repair system, the Cas protein is another important target for gene editing. If the promoter of the Cas9 protein is constitutive, the cleavage activity may not work in every cell. Huang et al. reported that 186.7 kb were deleted from the genome and the positive rates were over 95% when the Cas9 protein was expressed by an inducible promoter [25]. Therefore, the promoter of Cas9 protein was changed to P_ara_ (instead of constitutive promoter P_cas_ from *Streptococcus pyogenes*), an arabinose inducible stringent promoter from *E. coli*, which could be induced in *H. bluephagenesis* TD01 with least leak expression. The green fluorescent protein (GFP) was first used as reporter to investigate the induced expression intensity under 0–2% arabinose concentration (Fig. 4A). The highest ratio of GFP/OD_600_ was found when the arabinose concentration was 1.6%, and there are little differences in the ratio of GFP/OD_600_ from 0.8 to 1.4% arabinose concentration.

Meanwhile, we also found that the growth of *H. bluephagenesis* TD01 was inhibited when 1.6% arabinose concentration was used to induce Cas9 protein expression (data not shown). Therefore, the concentration of 0.2%, 0.4%, 0.6%, 0.8% and 1% arabinose were further used to determine the appropriate induction concentration by *phaC* gene deletion efficiency guided by two sgRNAs. A concentration of 1% arabinose was found to be the optimal induction con-centration for *phaC* gene deletion (Fig. 4B). We then deleted the 19 kb fragment (36,955–56,496 bp) using induced Cas9 expression. Forty-eight clones were selected for colony PCR and sequencing. The results showed that the fragment was successfully deleted guided by two sgRNAs with an efficiency of 12.5% with deletion length of approximate 19–23 kb (Table 4), whereas no deletion was found guided by two sgRNAs and only 1% deletion efficiency guided by three sgRNAs when Cas9 was constitutively ex-pressed before. This indicated that the CRISPR-Cas9-NHEJ (Mt) gene editing system is capable of deleting longer gene segments when Cas9 expression was induced.

**Table 4:** The deletion results of 19*–*50 kb fragments

Deletion fragment	Delete length (bp)	Colony number	Deletion efficiency
19 kb (36,955–56,496 bp)^*a*^	Wild type	42	12.5%
	19,000–23,000	6	
26 kb (692,569–718,967 bp)	Wild type	30	37.5%
	26,000–30,000	18	
50 kb (2,138,365–2,192,787 bp)	Wild type	33	31.3%
	50,000–55,000	15	

^*a*^ For 19 kb deletion, two sgRNAs with C/W location combination were used

**Fig. 5:**
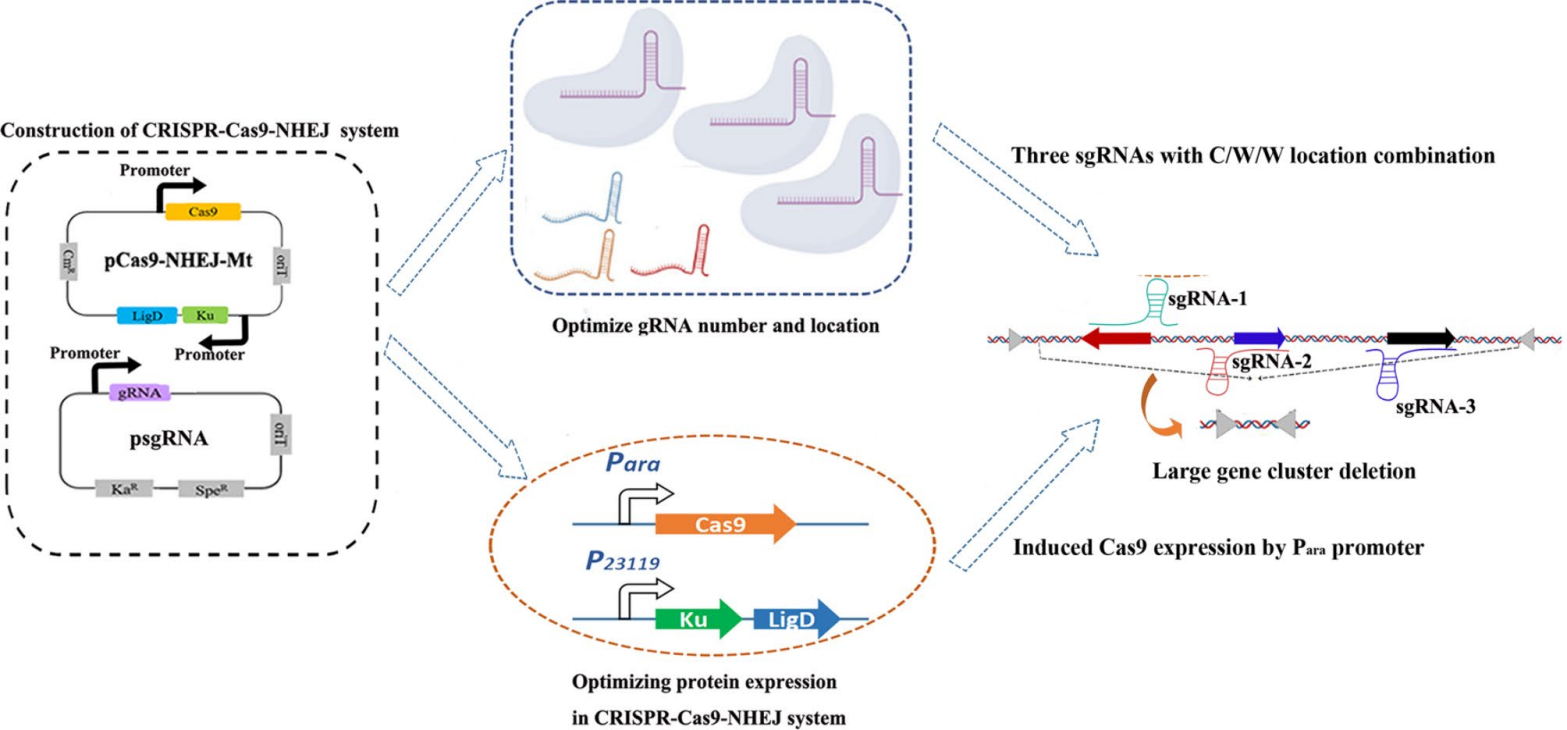
Schematic diagram for the entire procedure of construction and optimization of CRISPR-Cas9-NHEJ system for large gene cluster deletion in *H. bluephagenesis* TD01

Finally, using this optimized CRISPR-Cas9-NHEJ (Mt) gene editing system with induced Cas9 expression and three sgRNAs (Table S1) with C/W/W location combination, the 26 kb fragment (692,569–718,967 bp) and the 50 kb flagellar gene cluster (2,138,365–2,192,787 bp) deletion were conducted. For each experiment, forty-eight clones were randomly selected for colony PCR and sequencing. The results showed that the deletion efficiency of 26 kb fragment was 37.5% (Table 4), while that of 50 kb flagellar gene cluster reached 31.3% which is 2.5-fold (12.5%) higher than the improved CRISPR-Cas-HR gene editing system [4]. The deletion lengths were approximately 26–30 kb and 50–55 kb, respectively. Thus, the optimized CRISPR-Cas9-NHEJ (Mt) gene editing system could efficiently delete large fragments in different genome position and could be a powerful genome engineering tool in *Halomonas* for the efficient deletion of large DNA fragments. The entire procedure was illustrated in Fig. 5. Also, we found that the length of randomly deleted segments (up to 5 kb) at the cutting site for 15 kb, 18 kb, 19 kb, 26 kb, and 50 kb fragments were significantly smaller than that (up to 17 kb) for 4 kb, 7 kb, and 9 kb fragments (Tables 3 and 4). This indicated that the random deletion by NHEJ (Mt) system in *H. bluephagenesis* TD01 might be related to the length of target fragments.

## Discussion

NHEJ system is considered to be a major repair pathway for DNA double-stranded breaks [26]. The LigD protein of NHEJ system from *M. tuberculosis* and *Pseudomonas aeruginosa* has three functional domains: polymerase domain, nuclease domain, and ligase domain [27]. So, it tends to be prone to insertion and/or deletion mutations at the junctional site when repairing DSBs due to its nuclease and polymerase activity [22]. In this study, the exogenous NHEJ repair system derived from *M. tuberculosis* showed good efficiency for gene editing especially for large DNA fragment deletion in *H. bluephagenesis* TD01 combined with CRISPR-Cas9 system. However, these repair properties are different from in *E. coli*, although the same Cas9 protein and NHEJ (Mt) repair system were used. In *E. coli*, the CRISPR-Cas9-NHEJ (Mt) system could delete DNA fragment from 100 bp up to 17 kb with high efficiency [18], whereas in *H. bluephagenesis* TD01, this system could cause 10 bp up to 50 kb DNA fragment deletion. Another research reported that the CRISPR-Cas9 combined with NHEJ from *M. smegmatis* could achieve deletion of large DNA fragment up to 123 kb in *E. coli* [19], while this NHEJ system could not functionally work in *H. bluephagenesis* TD01. In addition, the CRISPR-Cas9-NHEJ (Mt) system in *E. coli* caused mainly deletion with different length, whereas the system in *H. bluephagenesis* TD01 caused not only deletion but also some base insertion up to more than 500 bp bases during repairing. These indicated that NHEJ systems have different repair properties in different microorganisms, so that the selection of exogenous NHEJ for use with CRISPR-Cas in different microorganisms needs to be experimentally verified.

In addition, NHEJ and HR are two major double-strand break repair pathways. In this study, both these two repair system existed when we introduced NHEJ (Mt) system into *H. bluephagenesis* TD01. It has been previously confirmed that the HR repair system inhibits the NHEJ repair system in *E. coli* [23]. RecX (inhibitory protein of RecA) overexpression resulted in a more-than-5-fold increase in the efficiency of Cas9-NHEJ-mediated genome editing of lacZ gene in *E. coli*. On the other hand, a 2.2 kb homologous recombination template was introduced into *Actinomycetes* and made higher efficiency, which indicated that HR system could also enhance the NHEJ system [20]. However in *H. bluephagenesis* TD01, both deletion and overexpression of the RecA protein almost completely inhibited the activity of the NHEJ repair system, which were different from the previous reports. There may be a balance between the native HR repair system and exogenous NHEJ repair system in *H. bluephagenesis* TD01 that needs to be investigated in the future. The relationship between HR and NHEJ may be a crucial point to improve the widespread application of the CRISPR-Cas-NHEJ gene editing system in prokaryotes. It may be possible to engineer appropriate promoters for the expression of different HR strengths to determine at which strength the NHEJ repair system is inhibited or enhanced.

Although we were able to successfully construct the CRISPR-Cas9-NHEJ (Mt) gene editing system and demonstrated that it can be used for gene knockout and deletion of large DNA fragment in *H. bluephagenesis* TD01, which may accelerate the use of *Halomonas* as chassis cells, many areas can be further improved and expanded. Other approaches, such as multi-stage induction, strict promoters and regulatory proteins could be potential strategies to improve the Mt-NHEJ repair activity. In some reports, it was shown that when inducible promoters of sgRNAs were used, multigene editing of three target genes was achieved simultaneously with the highest gene editing efficiency of 100% [28]. Therefore, in the future, knockouts of multiple genes could be performed by inducing several sgRNAs to achieve simultaneous multigene knockout in which the promoters of the sgRNAs are constitutive or inducible. We also tried multi-stage induction of Cas9 protein, and when the Cas9 was induced 4 and 5 times, the deletion efficiency of the 50 kb flagellar gene cluster reached 52.1% and 60.4%, respectively. The CRISPR-Cas9-NHEJ gene editing system can be used not only to delete large fragments, but also to insert fragments. In synthetic biology, genes are occasionally inserted to generate the desired product [29]. The CRISPR-Cas9-NHEJ gene editing system has been used for gene insertion in eukaryotes [30–35]. A plasmid is construct-ed for gene insertion and PAM sites are usually designed upstream and downstream of the inserted gene. Similar strategies and methods can be used to regulate the precise repair of the Mt-NHEJ repair system [36] and attempt to insert genes. However, the CRISPR-Cas9-NHEJ (Mt) gene editing system may repair the cut plasmid carrying the inserted fragment because of its non-selectivity for repair, resulting in low gene insertion efficiency, which should be initially avoided.

## Conclusions

In this study, we develop a CRISPR-Cas9 gene editing system combined with the NHEJ repair system from *M. tuberculosis* in *Halomonas* for the first time. By optimizing the sgRNA numbers, positions, and Cas9 expression, the improved CRISPR-Cas9-NHEJ (Mt) genome editing system was able to delete large DNA fragments of up to 50 kb quickly and efficiently compared with the CRISPR-Cas9-HR gene editing system. This genome editing system can be used to accelerate research on using *H. bluephagenesis* TD01 for chassis cell construction and further expand the application of the NHEJ repair system in prokaryotes.

## Methods

### Strains, media, and culture conditions

The *E. coli* DH5α strain was used for molecular cloning and plasmid manipulation. *E. coli* strain S17-1 was used as the host strain for joining transformation. *H. bluephagenesis* TD01 which was deposited in the China General Microbiological Culture Collection Center (CGMCC No. 4353) served as the genetic material for the editing experiments unless otherwise stated. The Luria-Bertani (LB) medium (10 g/L tryptone, 5 g/L yeast extract, 10 g/L NaCl) was used for the culture of *E. coli*. 60LB medium (10 g/L tryptone, 5 g/L yeast extract, 60 g/L NaCl) was used for the culture of *H. bluephagenesis* TD01. 20LB medium (10 g/L tryptone, 5 g/L yeast extract, 20 g/L NaCl) was used for conjugation transformation. The solid medium contained 15–20 g/L of agar. The working concentrations of chloromycetin, kanamycin and spectinomycin were 25 μg/mL, 50 μg/mL and 100 μg/mL, respectively. When necessary, L-arabinose (2–10 g/L) was added to the 60LB agar plate with chloromycetin and spectinomycin resistance to induce Cas9 protein expression.

### Construction of plasmids

The primers and plasmids used for plasmid construction in this study were listed in Tables S2 and S3, respectively. The plasmids related to the CRISPR-Cas9-NHEJ gene editing system were psgRNA, pCas9-P_cas_-NHEJ, and pCas9-P_ara_-NHEJ. The pCas9-P_cas_-NHEJ plasmid contained *cas9* gene with P_cas_ promoter from *S. pyogenes* and the Mt-NHEJ system (Ku and LigD genes) from *M. tuberculosis* H37Rv, Ms-NHEJ from *M. smegmatis* mc^2^ 155, or Bs-NHEJ from *B. subtilis* 168. The promoter of Ku and LigD proteins was the widely used constitutive promoter J23119 [28]. The pCas9-Mt-ZF and pCas9-Mt-ZR primers were used to amplify the *cas9*-containing vector, while the pCas9-Mt-PF and pCas9-Mt-PR primers were used to amplify the *ku* and *ligD* genes of Mt-NHEJ. *ku* and *ligD* from *M. smegmatis* mc^2^ 155 and *B. subtilis* 168 were directly synthesized. The pCas9-P_ara_-NHEJ plasmid was derived from the pCas9-P_cas_-NHEJ plasmid, whereas the arabinose inducible promoter P_ara_ from *E. coli* replaced the constitutive promoter P_cas_ for inducible expression of Cas9. P_ara_ was amplified using the pCas9Mt-ara-PF and pCas9Mt-ara-PR primers, and the vector was amplified using pCas9Mt-ara-ZF and pCas9Mt-ara-ZR primers. The Cas9 protein is usually expressed when large gene fragments are deleted.

psgRNA was constructed using the pSEVA241 plasmid as a backbone, and the J23119 promoter was used for sgRNA expression [3]. An important part of the psgRNA plasmid is the sgRNA sequence (a special spacer with a 20 bp base sequence). The spacer was selected on the website http://grna.ctegd.uga.edu/ and evaluated using the website http://www.oligoevaluator.com/. Normally, the spacer is inserted into the intermediate plasmid between the J23119 promoter and the sgRNA scaffold via plasmid amplification, in which the spacer is generally designed at the 5’ end of the amplification plasmid primer. *E. coli* DH5α can circularize linear plasmid because of the complementarity between spacer sequences. Two to three individual colonies were randomly selected for PCR verification and sequenced to verify whether the spacer was inserted correctly. The sgRNAs and target location used in this study were listed in Table S1. When using double sgRNAs guidance, the psgRNA-g12-ZF and psgR-NA-g12-ZR primers were used to amplify the vector and the psgRNA-g12-PF and psgRNA-g12-PR primers were used to amplify the fragment. When using three sgRNAs for guidance, the psgRNA-g123-ZF and psgRNA-g123-ZR primers were used to amplify the vector, and primers psgRNA-g123-PF and psgRNA-g123-PR were used to amplify the fragment.

DNA polymerases were purchased from TaKaRa (Dalian, China), and T4 DNA ligases from NEB (England). DNA purification, gel extraction and plasmid extraction kits were purchased from Omega (USA). Gibson Assembly kits for plasmid construction were purchased from Vazyme (Nanjing, China). DNA sequencing was performed by GENEWIZ (Suzhou, China).

### Gene fragment deletion

Conjugation transformation was used to transfer plasmids from *E. coli* S17-1 to *H. bluephagenesis* TD01. *E. coli* S17-1 was the donor cell and *H. bluephagenesis* TD01 was the recipient cell. The detailed experimental steps of conjugation transformation and gene knockout processes have been explained previously [3]. The constitutive P_cas_ promoter and inducible P_ara_ promoter were used for gene knockout. Cells containing the Cas9 protein and sgRNA plasmids were grown on 60LB solid plates with chloromycetin and spectinomycin. To induce Cas9 protein expression, individual colonies from the 60LB solid plates were streaked on chloromycetin and spectinomycin resistant 60LB solid plates containing 0.2–1% arabinose. Positive mutants were identified using colony PCR. The primer pairs used for colony PCR in this study were shown in Table S2. In addition, PCR products were sequenced to confirm successful gene or DNA fragment knockout.

### Plasmid curing and growth analysis

The procedure for plasmids curing was similar to that described in a previous study [3]. The pCas9-NHEJ (Mt) plasmid contains the *sacB* gene, and the *H. bluephagenesis* TD01 containing the pCas9-NHEJ (Mt) plasmid was streaked on 60LB agar plate containing 20% sucrose. Colonies growing on 60LB agar plate without chloramphenicol were the bacteria that had lost the plasmid.

A colony was selected and inoculated into 5 mL of liquid medium and precultured overnight at 37 °C and 200 rpm. Next, 1% of the seed culture solution was added to the fresh medium and incubated overnight the next day at 37 °C and 200 rpm. Samples were taken every 2 h to monitor the growth condition. All experiments were performed in triplicates.

### Electronic supplementary material

Below is the link to the electronic supplementary material.

**Additional file 1: Table S1**. The sequences of sgRNAs used for gene fragment deletion in this study. **Table S2**. Primers used for plasmids construction and colony PCR in this study; **Table S3**. Plasmids used in this study

## Data Availability

The datasets supporting the conclusions of this article are included in the manuscript and additional files.
