# Supplementary material for: CRISPR-Cas9 assisted non-homologous end joining genome editing system of Halomonas bluephagenesis for large DNA fragment deletion
Source: Microb Cell Fact. 2023 Oct 14;22:211. doi: 10.1186/s12934-023-02214-y (PMC10576340; doi:10.1186/s12934-023-02214-y)
Supplement: Supplementary file 1 — Additional file 1: Table S1. The sequences of sgRNAs used for gene fragment deletion in this study. Table S2. Primers used for plasmids construction and colony PCR in this study; Table S3. Plasmids used in this study [file 12934_2023_2214_MOESM1_ESM.docx]

**CRISPR-Cas9 Assisted Non-Homologous End Joining Genome Editing System of *Halomonas bluephagenesis* for large DNA fragment deletion**

Chunyan Liu^1,2^, Yaxin Yue^1,2^, Yanfen Xue^1^, Cheng Zhou^1,3,4*^, Yanhe Ma^1^,

^1^State Key Laboratory of Microbial Resources, Institute of Microbiology, Chinese Academy of Sciences, Beijing 100101, China;

^2^University of Chinese Academy of Sciences, Beijing 100049, China;

^3^College of Biochemical Engineering, Beijing Union University, Beijing 100023, China;

^4^Beijing Key Laboratory for Utilization of Biomass Wastes, Beijing 100023, China.

* Corresponding authors:

Cheng Zhou, E-mail: [zhoucheng@im.ac.cn](mailto:zhoucheng@im.ac.cn); zhoucheng@buu.edu.cn.

**Table S1.** The sequences of sgRNAs used for gene fragment deletion in this study.

| **Deletion gene fragment (location in genome)** | **sgRNA sequence (target location in fragment)** |
| --- | --- |
| *phaC* (3274665–3276515 bp) | tttccgtggtgggtgtgtat (3275393–3275412 bp) |
| *gabD3* (511151–512596 bp) | gctcgctcaatgtgcaggta (511861–511880 bp) |
| TD01-00059-61 (52204–55164 bp) | tgtcacgtcttcggaaaggc (53788–53807 bp) |
| TD01-01921-23 (3149319–2151802 bp) | aggccttcaggcagctcaga (2150506–2150525 bp) |
| *rfbD1* (604130–605026 bp) | caaactggaaggggatcaag (604627–604646 bp) |
| *rfbD2* (2205623–2206513 bp) | aacatgtggtacctgcccca (2205936–2205955 bp) |
| *phaC-*g1 (Crick) | gtgacagcaaatcctcaagc (3274766–3274785 bp) |
| *phaC*-g1 (Watson) | gggtggaaaatgccgtctca (3274674–3274693 bp) |
| *phaC*-g2 (Crick) | acgtcattgaaagcgctcct (3276439–3276458 bp) |
| *phaC*-g2 (Watson) | ggaagctcacctaacacctc (3276486–3276505 bp) |
| *phaC*-g3 (Crick) | tttccgtggtgggtgtgtat (3275578–3275597 bp) |
| *phaC*-g3 (Watson) | gttgagctactgtgtaggcg (3275657–3275676 bp) |
| 4 kb fragment (52204–56496 bp)-g1 (Crick) | tgctgcgccaagcaatgaaa (52305–52324 bp) |
| 4 kb fragment (52204–56496 bp)-g2 (Watson) | ccggcgaatgcgctatttaa (56057–56076 bp) |
| 7 kb fragment (49199–56507 bp)-g1 (Crick) | aatcgatagcgcgtataccg (49525–49544 bp) |
| 7 kb fragment (49199–56507 bp)-g2 (Watson) | ccggcgaatgcgctatttaa (56057–56076 bp) |
| 9 kb fragment (49407–58544 bp)-g1 (Crick) | gtctgggtttgccactagtc (47391–47410 bp) |
| 9 kb fragment (49407–58544 bp)-g2 (Watson) | ccggcgaatgcgctatttaa (56057–56076 bp) |
| 15 kb fragment (41405–56496 bp)-g1 (Crick) | ctgaccagcccaaaattcgc (41976–41995 bp) |
| 15 kb fragment (41405–56496 bp)-g2 (Watson) | ccggcgaatgcgctatttaa (56057–56076 bp) |
| 18 kb fragment (38533–56496 bp)-g1 (Crick) | gcgaaggtaggtaacatcca (38752–38771 bp) |
| 18 kb fragment (38533–56496 bp)-g2 (Watson) | ccggcgaatgcgctatttaa (56057–56076 bp) |
| 18 kb fragment (38533–56496 bp)-g3 (Watson) | gccgatgactacatcaccaa (47064–47083 bp) |
| 19 kb fragment (36955-56496 bp)-g1 (Crick) | atgtgcaattgccgcgctag (37020–37039 bp) |
| 19 kb fragment (36955-56496 bp)-g2 (Watson) | ccggcgaatgcgctatttaa (56057–56076 bp) |
| 26 kb fragment (692569–718967 bp)-g1 (Crick) | agcttcgctgactttgacgg (692630–692649 bp) |
| 26 kb fragment (692569–718967 bp)-g2 (Watson) | cattctcccgcctctgaatg (718912–718931 bp) |
| 26 kb fragment (692569–718967 bp)-g3 (Watson) | gttattcgtcgtgaccggag (705543–705562 bp) |
| 50 kb flagellum fragment (2138365–2192787 bp)-g1 (Crick) | aagaaagccgctaacgatgc (2138570–2138589 bp) |
| 50 kb flagellum fragment (2138365–2192787 bp)-g2 (Watson) | gacgcgtcgaactgctgtta (2192747–2192766 bp) |
| 50 kb flagellum fragment (2138365–2192787 bp)-g3 (Watson) | gcgaccaacagcacaaccaa (2163781–2163800 bp) |

**Table S2.** Primers used for plasmids construction and colony PCR in this study.

| **Name** | **Sequence** | **Description** |
| --- | --- | --- |
| pCas9-Mt-ZF | tatgcatggcgccctctgg | Application of vector fragment (8265 bp) |
| pCas9-Mt-ZR | caaagatctggactggctttctacgtggct |  |
| pCas9-Mt-PF | gccagtccagatctttgacagctagctcagt | Application of Mt-NHEJ (Ku and ligD genes) fragment (3272 bp) |
| pCas9-Mt-PR | agggcgccatgcataaaaaat |  |
| pCas9Mt-ara-ZF | tgtttctccatatggataagaaatactcaataggcttagatatcgg | Application of vector fragment (11113 bp) |
| pCas9Mt-ara-ZR | gaaaagtgctgcagggaaagccacg |  |
| pCas9Mt-ara-PF | ccctgcagcacttttcggggaaatgtgttatgac | Application of P_ara_ fragment (1229 bp) |
| pCas9Mt-ara-PR | tttcttatccatatggagaaacagtagagagttgcga |  |
| psgRNA-g12-ZF | ctcgcagagcaggattcccgttaccgggttgg | Application of vector fragment (5207 bp) |
| psgRNA-g12-ZR | tagcacaatacctaggactggcagcagattac |  |
| psgRNA-g12-PF | ttctgcgcgtaatctgctgccagtcctaggtattgtgctagcg | Application of fragment of three sgRNAs (263 bp) |
| psgRNA-g12-PR | tcttgagtccaacccggtaacgggaatcctgctctgc |  |
| psgRNA-g123-ZF | ggccagcgcgcgaattcgagct | Application of vector fragment (5409 bp) |
| psgRNA-g123-ZR | taggactgcgcggcttaggcgg |  |
| psgRNA-g123-PF | agccgcgcagtcctaggtattgtgctagcggc | Application of fragment of three sgRNAs (202 bp) |
| psgRNA-g123-PR | attcgcgcgctggccgtaggcgcg |  |
| phaC-F | atgctgtcagggtggaaaat | Used for colony PCR to validate deletion of *phac* gene |
| phaC-R | ttacgacgcgggaagctcac |  |
| rfbC1-F | actcgtcgcttccgtagctt | Used for colony PCR to validate deletion of *rfbC1* gene |
| rfbC1-R | atgaacatcctcatcaccgg |  |
| rfbC2-F | gtgaagttgctgatactaga | Used for colony PCR to validate deletion of *rfbC2* gene |
| rfbC2-R | tcaagccaactctcctgtcc |  |
| gabD3-F | ttacaggccgccaacacaga | Used for colony PCR to validate deletion of *gabD3* gene |
| gabD3-R | atgagtaagttacccgatat |  |
| TD01-01921-23-F | atgcgtattagtacggtcac | Used for colony PCR to validate deletion of TD01-01921-23 |
| TD01-01921-23-R | atgaccccggaaatggtgat |  |
| TD01-00059-61-F | tcacggcatcgttctcgacc | Used for colony PCR to validate deletion of TD01-00059-61 |
| TD01-00059-61-R | atggatcagacgaccattgaga |  |
| 15 kb-F | cgctgattgtcactaactgc | Used for colony PCR to validate deletion of 15 kb fragment (2000 bp upstream or downstream of the cutting sites) |
| 15 kb-R | cagggaacgggcctacaatt |  |
| 18 kb-F | cagcgttgtacgcagtgcgt | Used for colony PCR to validate deletion of 18 kb fragment (2000 bp upstream or downstream of the cutting site) |
| 18 kb-R | cagggaacgggcctacaatt |  |
| 19 kb-F | atgactcatcacaacaacgc | Used for colony PCR to validate deletion of 19 kb fragment (2000 bp upstream or downstream of the cutting site) |
| 19 kb-R | cagggaacgggcctacaatt |  |
| 26 kb-F | tgcctctttgtggcgcatta | Used for colony PCR to validate deletion of 26 kb fragment (2000 bp upstream or downstream of the cutting site) |
| 26 kb-R | Tttgccctaatgcggcttta |  |
| 50 kb-F | tagctgggaggctttgaatc | Used for colony PCR to validate deletion of 50 kb fragment (2000 bp upstream or downstream of the cutting site) |
| 50 kb-R | ctttacgctcgcgctccagg |  |

**Table S3.** Plasmids used in this study.

| Plasmid | Relevant characteristics | Source |
| --- | --- | --- |
| pCas9-NHEJ (Mt) | pQ08-derived plasmid; Cas9 and Mt-NHEJ repair system is constitutive expression; Chloramphenicol resistance. | This study |
| pCas9-NHEJ (Ms) | pQ08-derived plasmid; Cas9 and Ms-NHEJ repair system is constitutive expression. Chloramphenicol resistance. | This study |
| pCas9-NHEJ (Bs) | pQ08-derived plasmid; Cas9 and Bs-NHEJ repair system is constitutive expression; Chloramphenicol resistance. | This study |
| pCas9-P_ara_-NHEJ (Mt) | Arabinose induces P_ara_ promoter expression of Cas9 protein; Mt-NHEJ repair system is constitutive expression; Chloramphenicol resistance. | This study |
| psgRNA | gRNA is constitutive expression; Spectinomycin and Kanamycin resistance. | [3] |
| psgRNA-*phaC* | psgRNA-derived plasmid contains gRNA sequence targeting the *phaC* gene; Spectinomycin and Kanamycin resistance. | This study |
| psgRNA-J23119-*recA* | psgRNA-derived plasmid contains gRNA sequence targeting the *phaC* gene and J23119 promoter expresses RecA protein; Spectinomycin and Kanamycin resistance. | This study |
